# *Akkermansia muciniphila* phospholipid induces homeostatic immune responses

**DOI:** 10.1038/s41586-022-04985-7

**Published:** 2022-07-27

**Authors:** Munhyung Bae, Chelsi D. Cassilly, Xiaoxi Liu, Sung-Moo Park, Betsabeh Khoramian Tusi, Xiangjun Chen, Jaeyoung Kwon, Pavel Filipčík, Andrew S. Bolze, Zehua Liu, Hera Vlamakis, Daniel B. Graham, Sara J. Buhrlage, Ramnik J. Xavier, Jon Clardy

**Affiliations:** 1 Department of Biological Chemistry and Molecular Pharmacology, Harvard Medical School, Blavatnik Institute, Boston, MA USA; 2 Department of Cancer Biology and the Linde Program in Cancer Chemical Biology, Dana-Farber Cancer Institute, Boston, MA USA; 3 Broad Institute of MIT and Harvard, Cambridge, MA USA; 4 Department of Molecular Biology, Massachusetts General Hospital, Harvard Medical School, Boston, MA USA; 5 Center for Computational and Integrative Biology, Massachusetts General Hospital, Harvard Medical School, Boston, MA USA; 6 Biochemistry Department, School of Biomedical Sciences, University of Otago, Dunedin, New Zealand; 7 SBGrid Consortium, Harvard Medical School, Blavatnik Institute, Boston, MA USA; 8 Present Address: College of Pharmacy, Gachon University, Incheon, South Korea; 9 Present Address: Natural Product Informatics Research Center, Korea Institute of Science and Technology (KIST), Ganeung, South Korea

**Keywords:** Bacterial host response, Biochemistry

## Abstract

Multiple studies have established associations between human gut bacteria and host physiology, but determining the molecular mechanisms underlying these associations has been challenging^1–3^. *Akkermansia muciniphila* has been robustly associated with positive systemic effects on host metabolism, favourable outcomes to checkpoint blockade in cancer immunotherapy and homeostatic immunity^4–7^. Here we report the identification of a lipid from *A. muciniphila*’s cell membrane that recapitulates the immunomodulatory activity of *A. muciniphila* in cell-based assays^8^. The isolated immunogen, a diacyl phosphatidylethanolamine with two branched chains (a15:0-i15:0 PE), was characterized through both spectroscopic analysis and chemical synthesis. The immunogenic activity of a15:0-i15:0 PE has a highly restricted structure–activity relationship, and its immune signalling requires an unexpected toll-like receptor TLR2–TLR1 heterodimer^9,10^. Certain features of the phospholipid’s activity are worth noting: it is significantly less potent than known natural and synthetic TLR2 agonists; it preferentially induces some inflammatory cytokines but not others; and, at low doses (1% of EC_50_) it resets activation thresholds and responses for immune signalling. Identifying both the molecule and an equipotent synthetic analogue, its non-canonical TLR2–TLR1 signalling pathway, its immunomodulatory selectivity and its low-dose immunoregulatory effects provide a molecular mechanism for a model of *A. muciniphila’*s ability to set immunological tone and its varied roles in health and disease.

## Abstract (web summary)

Overall, this study describes the molecular mechanism of a druggable pathway that recapitulates in cellular assays the immunomodulatory effects associated with *Akkermansia muciniphila,* a prominent member of the gut microbiota.

## Main

There are numerous correlations between gut microbes and host responses, but the responsible molecules and mechanisms are largely unknown^1–3^. *Akkermansia muciniphila*, a recently discovered member of the gut microbiome, appears prominently in these correlations^11^. This Gram-negative obligate anaerobe comprises approximately 3% of healthy human gut populations and is primarily known as a phylogenetic outlier that degrades intestinal mucin, the mucus layer separating the epithelial cells forming the intestinal wall from the intestine’s contents^12^. Its abundance is inversely correlated with inflammatory bowel disease and type 2 diabetes, but positively correlated with responses to programmed cell-death 1 (PD-1) or programmed cell-death-ligand 1 (PD-L1) checkpoint inhibitors in cancer immunotherapy^6,7,13^. These correlations gained additional importance when a recent report identified *A. muciniphila*’s unusual ability to induce intestinal adaptive immune responses during homeostasis in a subset of T cells^5^.

In an earlier study to identify immunoregulatory small molecules from gut microbes, we used an unbiased functional assay using cytokine release from murine bone-marrow-derived dendritic cells (mBMDCs) in response to fractionated bacterial extracts^8^. Dendritic cells, which are part of the innate immune system, detect pathogen-associated molecules and relay information to the adaptive immune system through the release of cytokines. We reasoned that the same approach would identify immunogens produced by *A. muciniphila*.

**Fig. 1:**
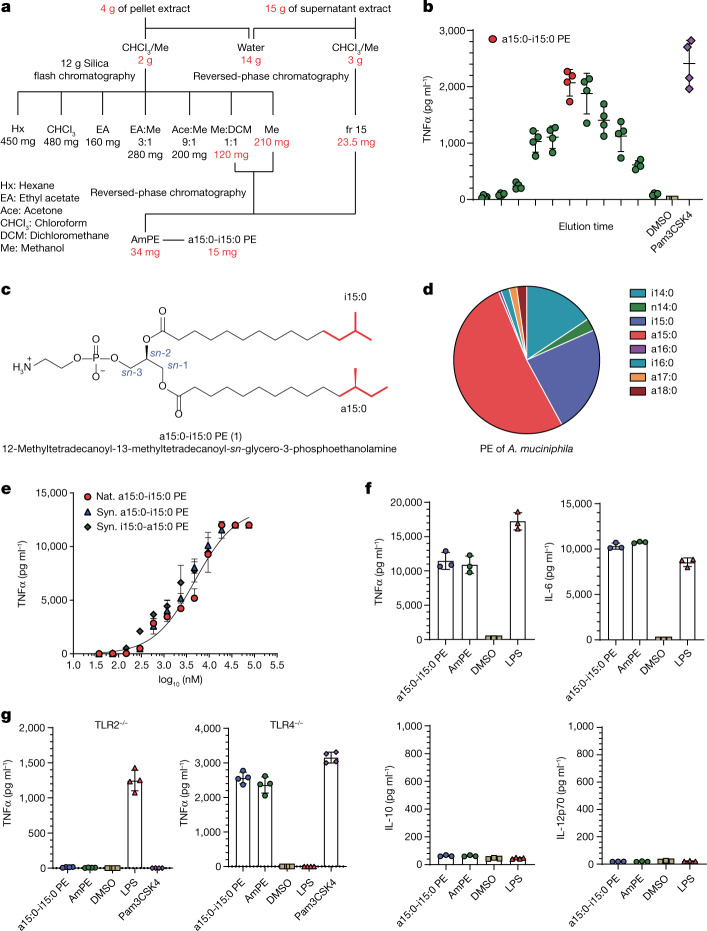
Structural and functional analysis of *A. muciniphila* PE. **a**, Flow diagram for fractionation of *A. muciniphila* PE. Amounts in active fractions are shown in red. FA composition of PE fraction also shown. **b**, TNFα production by mBMDCs treated with *A. muciniphila* lipid extract fractions as measured by ELISA. The fraction indicated in red was used for structural characterization. Pam3CSK4 was used as a control agonist. Data are presented as mean values ± s.d. of technical replicates (*n* = 4). **c**, The structure of a15:0-i15:0 PE. **d**, The relative abundance of FAs in *A. muciniphila* PE. **e**, Dose response of TNFα production by mBMDCs treated with natural (Nat.) and synthetic (Syn.) a15:0-i15:0 PE lipids as measured by ELISA. Data are presented as mean values ± s.d. of technical replicates (*n* = 4). **f**, a15:0-i15:0 PE and complete PE (AmPE) trigger release of TNFα and IL-6 but not IL-10 or IL-12p70 from mBMDCs, as measured by flow cytometry. LPS was used as a control. Data are presented as mean values ± s.d. of technical replicates (*n* = 3). **g**, TNFα release is lost in TLR2 knockout mBMDCs but not in TLR4 knockout mBMDCs as measured by ELISA. Pam3CSK4 was used as a TLR2 control agonist, and LPS was used as a TLR4 control agonist. Data are presented as mean values ± s.d. of technical replicates (*n* = 4). All experiments were repeated independently at least twice with similar results. DMSO, dimethyl sulfoxide.

**Extended Data Fig. 1:**
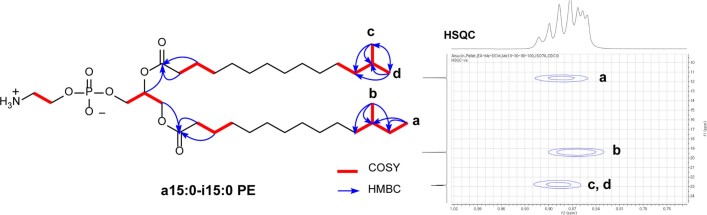
Characterization of a15:0-i15:0 PE. Key COSY, HSQC, and HMBC correlations of a15:0-i15:0 PE. All experiments were repeated independently at least twice with similar results.

To conduct a comprehensive survey for immunogens, both the cell pellet and supernatant from *A. muciniphila* BAA-835 cultures were assayed for their ability to induce cytokine release from mBMDCs (Fig. 1a)^8^. The crude lipid fraction from a small initial culture produced significant TNFα (tumour necrosis factor A) induction (Fig. 1b). Large-scale culturing (128 l) led to a combined lipid extract (19 g), which, upon further chromatographic separations with normal- and reversed-phase chromatography, led to a single active fraction with robust TNFα induction (Fig. 1a). The active fraction was a mixture of closely related molecules with a major component (15 mg) (Fig. 1b). Mass spectroscopic analysis indicated a molecular formula of C_35_H_71_NO_8_P, suggesting a phospholipid, and preliminary ^13^C and ^1^H nuclear magnetic resonance (NMR) analysis identified a phosphatidylethanolamine (PE), the dominant membrane phospholipid in most bacteria^14^. PEs have a glycerol core, a polar phosphoethanolamine head group at the *sn*-3 position, and two fatty-acid (FA) esters attached to the *sn*-1 and *sn*-2 positions (Fig. 1c). Additional NMR analysis revealed that both chains had methyl branches (Fig. 1c and Extended Data Fig. 1). One acyl chain had a terminal *iso* branch, and the other had a terminal *anteiso* branch, meaning that the methyl groups were on positions 12 and 13 of a 14-carbon FA chain (Fig. 1c). The order of the acyl groups was determined by selective hydrolysis to preferentially liberate the FA attached at the *sn*-2 position. The active molecule’s chemical name is 12-methyltetradecanoyl-13-methyltetradecanoyl-*sn*-glycero-3-phosphoethanolamine, which is a15:0-i15:0 PE in standard lipid nomenclature. We did not find producers of a15:0-i15:0 PE in frequently encountered gut microbes nor in gut microbes with reported immunomodulatory effects^2^. Membrane lipids reflect both evolutionary history and current environment. *A. muciniphila*, the only member of verrucomicrobia in the gut microbiota, is a phylogenetic outlier specialized for life in the mucin layer. Metabolomic analysis, phylogenetic placement and a distinctive microenvironment all support a singular association of *A. muciniphila* with a15:0-i15:0 PE.

The active fraction contained all the PEs produced by *A. muciniphila*; the later eluting fractions were triglycerides and the earlier eluting fractions were diacylglycerides with different head groups. The PE FAs were dominated (92%) by relatively short, branched-chain fatty acids (BCFAs): a15:0 (52%), i15:0 (24%) and i14:0 (16%) (Fig. 1d). Small amounts of a17:0 and i16:0 were also present. Bacteria make BCFAs to increase membrane fluidity, the same function unsaturated FAs have in animals^15^. *Anteiso* FAs increase fluidity more than *iso* FAs and *iso* FAs increase fluidity over normal FAs^16^. BCFAs are common in bacteria, including many pathogens, but they can be produced by humans at low levels^17^. Interestingly, BCFAs in human serum, independent of a connection with *A. muciniphila* or any other bacteria, have been strongly associated with human health, especially an anticorrelation with developing type 2 diabetes^18,19^.

**Extended Data Fig. 2:**
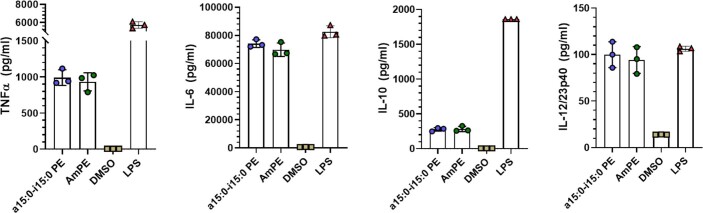
Human-cell activation by PE of *A. muciniphila*. a15:0-i15:0 PE induces TNFα, IL-6, IL-10, and IL-12/23p40 from human myeloid cells as measured by flow cytometry. Data are presented as mean values ± s.d. of technical replicates (*n* = 3). Experiments were repeated independently at least twice with similar results.

The active compound (a15:0-i15:0 PE) was the major component (approximately 50%) of *A. muciniphila*’s lipid membrane and had a robust dose–response curve for induction of TNFα (Fig. 1e). In addition to dramatically upregulating TNFα release, it promoted the release of IL-6 (interleukin 6), but not IL-10 or IL-12p70 (Fig. 1f and Extended Data Fig. 2). Dendritic cells typically respond to bacterial metabolites through the pathogen-associated molecular pattern (PAMP) receptors, toll-like receptor 2 (TLR2) and toll-like receptor 4 (TLR4)^9,20^. Receptor specificity was established by generating mBMDCs from both *tlr*2^−/−^ and *tlr*4^−/−^ mice and using them along with wild-type cells in the cytokine induction assay^8^. PEs active in wild-type cell assays produced no TNFα induction in mBMDCs from *tlr*2^−/−^ mice but showed robust TNFα induction in mBMDCs generated from *tlr*4^−/−^ mice (Fig. 1g). Previous reports attributed *A. muciniphila*’s immunomodulatory activity to a membrane-associated protein (Amuc_1100) signalling through TLR2^21,22^. Additional publications supporting the protein’s role in maintaining the intestinal mucosal barrier have also appeared^23^. Even though a15:0-i15:0 PE was the only active molecule detected in our study, other immunomodulatory contributors are a distinct possibility^24^.

**Fig. 2:**
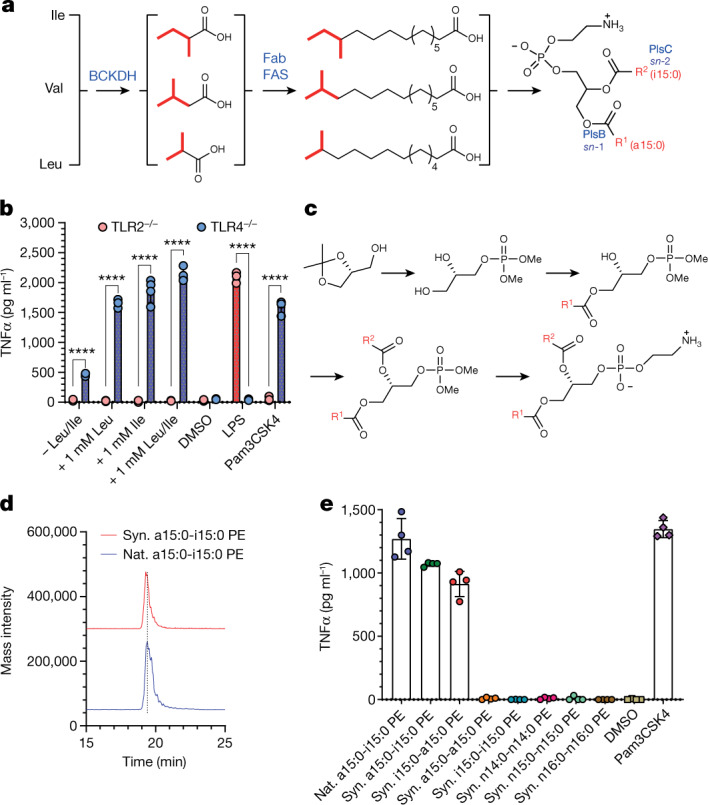
Biosynthesis and laboratory synthesis of *A. muciniphila* PE. **a**, Key genes involved in the putative biosynthetic pathway for *A. muciniphila* BAA-835 PE. **b**, Leucine or isoleucine feeding increases TNFα induction by *A. muciniphila* in a TLR2-dependent fashion as measured by ELISA. Pam3CSK4 and LPS were used as controls. Data are presented as mean values ± s.d. of technical replicates (*n* = 4). Unpaired *t*-test with two-tailed *P* value; ****P < 0.0001. **c**, Outline of synthetic scheme for a15:0-i15:0 PE and analogues. **d**, Overlay of mass spectrometric data from the natural and synthetic a15:0-i15:0. **e**, TNFα induction by natural and synthetic a15:0-i15:0 PE. a15:0-i15:0 PE induces production in mBMDCs, whereas n14:0-n14:0, n15:0-n15:0, n16:0-n16:0, a15:0-a15:0 and i15:0-i15:0 PE have no detectable TNFα induction, as measured by ELISA. i15:0-a15:0, the positional isomer, shows partial induction. Pam3CSK4 and LPS were used as controls. Data are presented as mean values ± s.d. of technical replicates (*n* = 4). All experiments were repeated independently at least twice with similar results. Ile, isoleucine; Val, valine; Leu, leucine.

**Extended Data Fig. 3:**
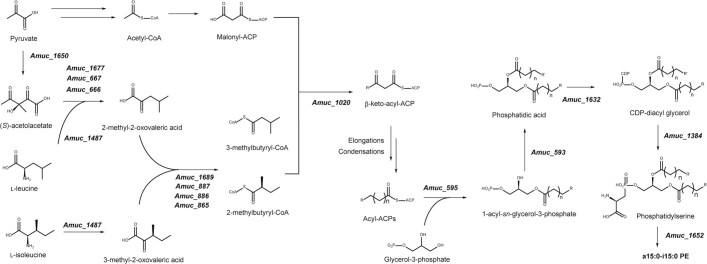
Biosynthetic pathway of a15:0-i15:0 PE in *A. muciniphila*. Proposed biosynthesis of a15:0-i15:0 PE in *A. muciniphila* BAA-835.

**Extended Data Fig. 4:**
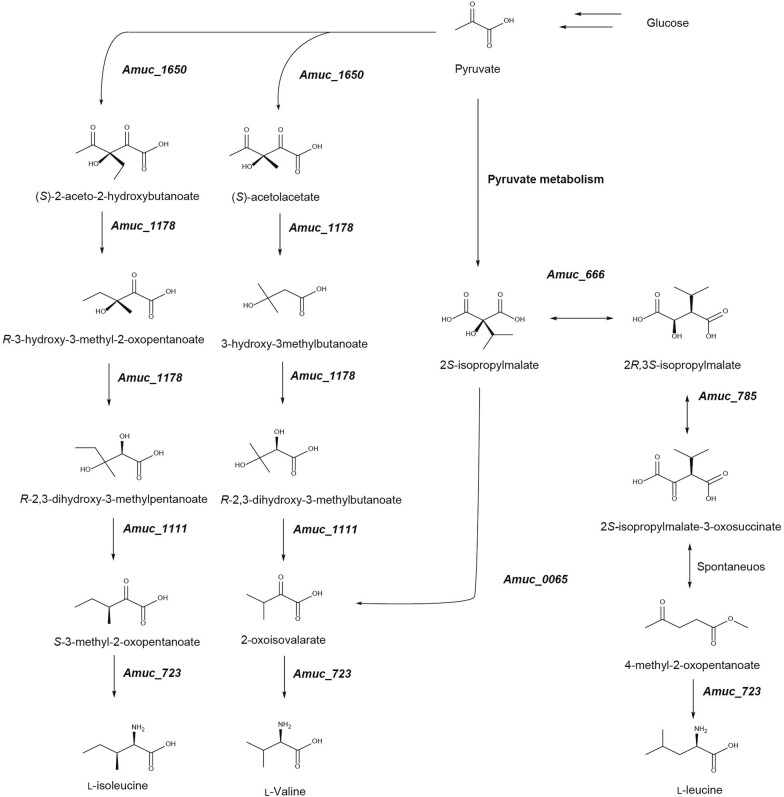
Biosynthetic pathways of branched-chain amino acids in *A. muciniphila*. *De novo* biosynthesis of l-leucine, l-isoleucine, and l-valine in *A. muciniphila* BAA-835.

With the identification of a15:0-i15:0 PE as an immunomodulatory molecule and TLR2 as its cognate receptor, we examined the pathway and regulation of PE biosynthesis by *A. muciniphila* and the laboratory synthesis of a15:0-i15:0 PE (Fig. 2). Bacterial PE biosynthesis has three distinct stages (Fig. 2a)^25^. In the first stage, the branched-chain amino acids (BCAAs) isoleucine (Ile), leucine (Leu) and valine (Val) are converted to branched-chain carboxylic acids by the branched-chain alpha-keto acid dehydrogenase complex (BCKDH)^25^. These acids are shuttled into the FA synthase (FAS) cycle by FabH (3-oxoacyl-[acyl-carrier-protein] synthase 3) where they become the tail end of an FA chain through the repetitive addition of two-carbon units^25^. After elongation, the BCFAs are added to the *sn*-1 and *sn*-2 positions of glycerol-3-phosphate by the enzymes PlsB and PlsC, respectively. The composition of PEs is regulated by numerous factors from the availability of BCAAs to the selectivity of PlsB and PlsC. Finally, the phosphate head group is elaborated into a PE head group^25^. The *A. muciniphila* genome has genes that encode the enzymes for every step of the BCAA to PE pathway just described (Extended Data Fig. 3). In addition, *A. muciniphila* has the genes for the de novo synthesis of BCAAs from glucose (Extended Data Fig. 4). The general pathway outlined above is supported by feeding experiments (Fig. 2b). Lipid extracts from *A. muciniphila* grown in minimal media supplemented with Leu and/or Ile used to treat mBMDCs led to significant increases in TNFα release in a TLR2-dependent fashion. Experiments in rich (brain heart infusion (BHI)) media showed similar but smaller increases.

We synthesized candidate PEs to independently confirm our identification, provide additional material for biological testing, eliminate the possibility of natural contaminants and explore structure–activity relationships (SARs). The synthesis, which is outlined in Fig. 2c, began with a commercially available protected chiral glycerol. With the future *sn*-2 and *sn*-3 positions blocked, the future *sn*-3 position was converted to a protected phosphate. The hydroxyl groups at *sn*-2 and *sn*-3 were deprotected, and the acyl groups were added in a stepwise fashion, taking advantage of the greater reactivity at the *sn*-1 position to install this acyl chain first. The a15:0 carboxylic acid used in the synthesis had the stereochemistry appropriate for natural Ile. The synthetic a15:0-i15:0 PE had identical spectroscopic, chromatographic and biological properties to the natural molecule (Figs. 1e and 2d,e).

**Extended Data Fig. 5:**
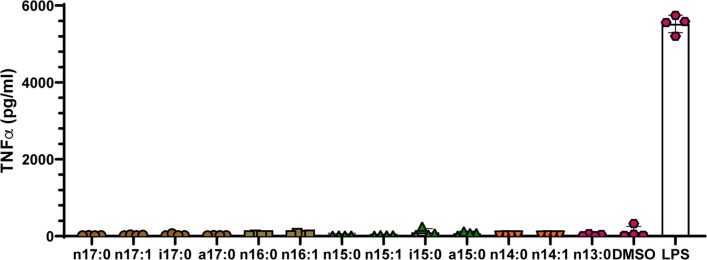
TNFα activation of straight chain fatty acids. TNFα production by mBMDCs treated with single chain fatty acids (from C17:0 to C13:0) as measured by ELISA. LPS was used as a control agonist. Data are representative of at least two independent experiments and show mean values ± s.d. of technical replicates (*n* = 4).

In addition to confirming the order of the acyl chains and two stereochemical issues, the synthetic scheme allowed a small library of natural and synthetic FAs and diacyl PEs to be assembled. The library was assayed to establish a preliminary SAR for the *A*. *muciniphila* lipids and their component parts. First, we established that FAs only activate TLR2 in the context of a diacyl PE, as none has any detectable activity on its own (Extended Data Fig. 5). This result is consistent with the *tlr*2^−/−^ mBMDC analysis (Fig. 1g). The diacyl PE library members revealed a surprisingly strict set of structural requirements (Fig. 2e): (1) methyl branches are essential for TNFα induction, as the three PE analogues where both acyl groups have straight chains (n14:0, n15:0 and n16:0) had no detectable activity; (2) the two acyl chains must be different, as a15:0-a15:0 PE and i15:0-i15:0 PE had no detectable activity; and (3) positional order appears to play a minor role, as a15:0-i15:0 PE and i15:0-a15:0 PE were essentially equipotent. We did not detect i15:0-a15:0 PE in natural samples.

**Fig. 3:**
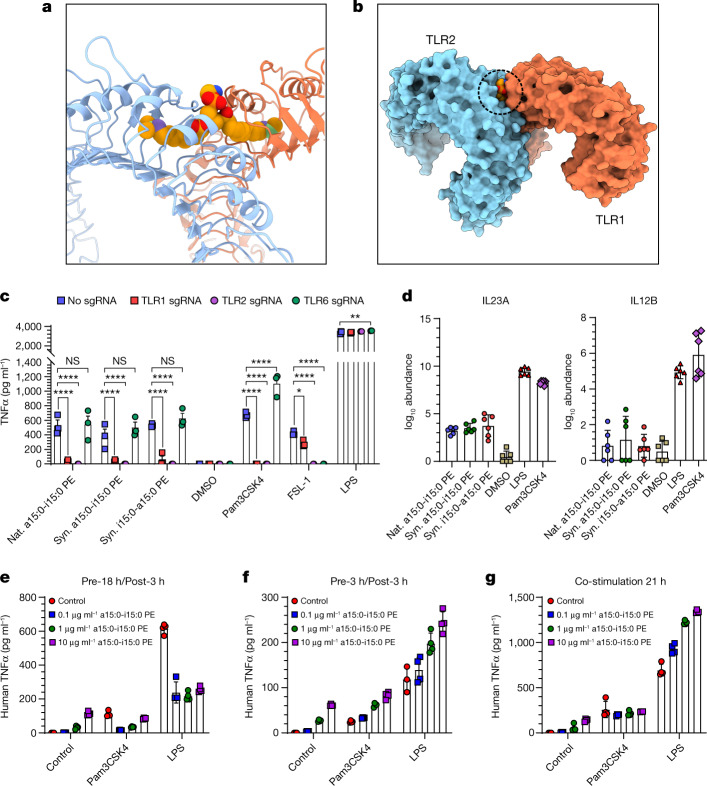
TLR2–TLR1 binding model and T cell activation by a15:0-i15:0 PE. **a**, View of the TLR2–TLR1 complex from the Protein Data Bank (PDB ID 2z7x) with the modelled a15:0-i15:0 PE ligand in the ‘bridging’ conformation, showing the branches with C13 coloured green and C12 purple. **b**, An overview of the modelled TLR2–TLR1-a15:0-i15:0 PE complex in the surface representation. The dashed circle indicates the buried lipid head group. **c**, TLR1 and TLR2 are required for natural and synthetic *A. muciniphila* lipids to induce TNFα production in human monocyte-derived dendritic cells (MDDCs). The production of TNFα was measured by ELISA 18 h after adding natural or synthetic *A. muciniphila* lipids, Pam3CSK4, FSL-1 or LPS to cell culture media of human MDDCs following nucleofection. **d**, IL-23A and IL-12B induction by natural and synthetic a15:0-i15:0 PE lipids. **e**–**g**, Effects of treatment of human MDDCs with a15:0-i15:0 PE in combination with Pam3CSK4 or LPS. With long (18 h) delay times, low doses of a15:0-i15:0 PE suppress immune responses to Pam3CSK4 and moderate immune responses to LPS (**e**). Both effects disappear with shorter delay times (3 h in **f** or none in **g**). LPS and Pam3CSK4 were used at final concentrations of 100 ng ml^−1^. Data in **c** (*n* = 3), **d** (*n* = 6) and **e**–**g** (*n* = 4) are representative of two independent experiments, showing mean values ±  s.d. *P* values in **a** were calculated by two-way analysis of variance. *P *<* 0.05; **P < 0.001; ****P < 0.0001; NS, not significant.

SAR studies on TLR2 ligands invariably focus on the head group that protrudes from the membrane-bound receptor. The conventional view of TLR2 signalling relegates the lipid chains to providing hydrophobic anchors for a protruding head group that regulates receptor activation^9,26,27^. This view is supported by several structural studies on TLR2 receptors with bound ligand and SAR studies^26,28,29^. The extracellular part of TLR2 is a horseshoe-shaped, leucine-rich repeat with a long hydrophobic tunnel that binds two acyl chains (Fig. 3a,b). TLR2 typically requires formation of a heterodimer with either TLR1 or TLR6 for immune signalling^9,28,29^. CRISPR–Cas knockdowns of TLR6 and TLR1 showed that a TLR2–TLR1 heterodimer is required for TNFα induction, which is a surprising result for a diacyl lipid (Fig. 3a,c)^9,28,29^. The requirement for a non-canonical TLR2–TLR1 heterodimer indicates that the two acyl chains of a15:0-i15:0 PE occupy binding pockets in two different proteins, one in TLR2 and one in TLR1, forming an atypical signalling heterodimer with a buried head group (Fig. 3b and Supplementary Video 1). There are other TLR2 agonists that form TLR2–TLR1 heterodimers, and at least two of them (the synthetic molecules diprovocim and CU-T12-9) were developed as adjuvants for cancer immunotherapy, whereas another (polysaccharide A from *Bacteroides fragilis*) is produced by a member of the gut microbiome and associated with IL-10 production^30–33^. There is an important difference in potency between the synthetic agonists and a15:0-i15:0 PE: EC_50_ values of pmol l^−1^ versus µmol l^−1^. A similar difference in immunogenicity has been noted in a study of immunomodulatory sphingolipids from *B*. *fragilis*^24^.

**Extended Data Fig. 6:**
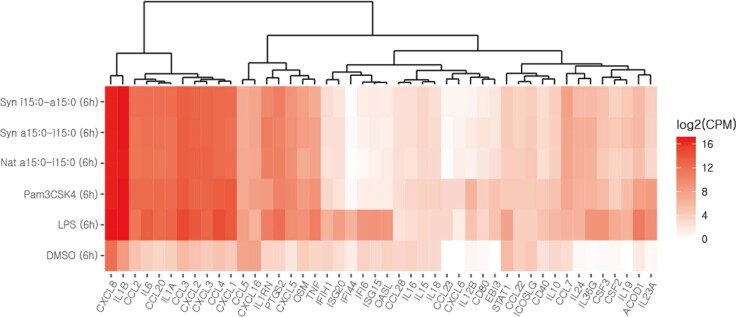
Heat-map of human derived monocyte activation by natural and synthetic *A. muciniphila* lipids. Monocytes were purified from peripheral blood by negative selection with magnetic beads. Cells were immediately placed in culture and stimulated with the indicated stimuli for 6 h. After stimulation, cells were lysed for mRNA extraction and library preparation by SmartSeq2 for RNA sequencing. Monocytes were processed from two independent donors in technical triplicate. The final concentration of bacterial lipids was 50 μg/ml. Pam3CSK4, FSL-1, and LPS at a final concentration of 100 ng/ml were used as controls.

To complete this initial phase of our study, we sought to connect the active lipids from *A*. *muciniphila* to the selective cytokine responses of specific human immune cell lineages^5^. Human monocytes purified from peripheral blood were cultured and stimulated with natural and synthetic TLR2 agonists for 6 h, after which mRNA was extracted and sequenced (Extended Data Fig. 6). The a15:0-i15:0 PE induced pro-inflammatory cytokines such as TNFα and IL-6 comparably with lipopolysaccharide (LPS) and Pam3CSK4, albeit at higher doses, but was significantly less effective at inducing IL-23, a heterodimer of IL-23A and IL-12B (Figs. 1f and 3d and Extended Data Figs. 2 and 6). The IL-23/T-helper-17 cell (T_h_17) immune axis is a major inflammatory pathway, and its therapeutic regulation is an important research target^34^.

**Extended Data Fig. 7:**
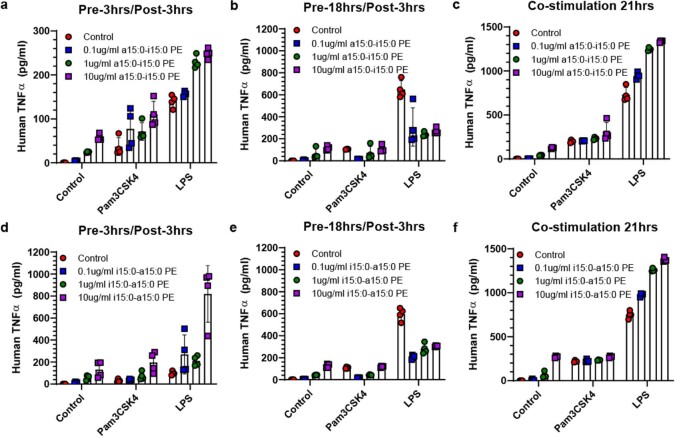
Effects of treatment with synthetic *A. muciniphila* lipids in combination with Pam3CSK4 or LPS on human monocyte-derived dendritic cells. With long (18 h) delay times, low doses of synthetic **a**–**c**, a15:0-i15:0 PE or **d**–**f**, i15:0-a15:0 PE suppress immune responses to Pam3CSK4 and moderate immune responses to LPS. Both effects disappear with shorter delay times (3 h or none). LPS and Pam3CSK4 were used at final concentrations of 100 ng/mL. Data are presented as mean values ± s.d. of technical replicates (n = 4). Experiments were repeated independently at least twice with similar results.

Next, we investigated the effects of a15:0-i15:0 PE on other immunogens by treating human monocyte-derived dendritic cells (which were differentiated by granulocyte-macrophage colony-stimulating factor (GM-CSF) and IL-4) with a15:0-i15:0 PE and the model agonists Pam3CSK4 (to stimulate TLR2–TLR1) and LPS (to stimulate TLR4). Variables were dose, duration (6 h or 21 h) and timing (cotreatment or sequential treatment). A particularly informative set of data came from the 21 h sequential treatment study in which a low dose of a15:0-i15:0 PE (0.15 µmol l^−1^, approximately 1% of EC_50_) was followed 18 h later by the addition of known agonists. This treatment regimen completely suppressed TNFα release (Fig. 3e and Extended Data Fig. 7). The suppressive effect was not seen with shorter periods between the lipid and agonist treatments (6  h and co-stimulation studies) nor with higher doses of lipid (Fig. 3f,g and Extended Data Fig. 7). These results support a model in which low doses of a15:0-i15:0 PE and delayed stimulation reset the cellular activation threshold and moderate other cellular immune responses, as indicated by the LPS response, which was reduced but not in a lipid-dependent fashion (Fig. 3e). Low dose and delayed stimulation reflect likely in vivo conditions. Larger doses and shorter times produce the expected dose-dependent response (Figs. 1e and 3f,g and Extended Data Fig. 7).

## Conclusion

Since its discovery, multiple lines of investigation have indicated that *A*. *muciniphila* plays a considerable role in regulating human immune responses in a variety of contexts^4–7^. Our study indicates that *A. muciniphila’s* immunomodulatory activity can be replicated by a diacyl PE, a15:0-i15:0 PE, a lipid that is not noticeably different from other diacyl PEs forming the cell membranes of most bacteria found in the human gut^14^. Because of its generic structure, its remarkable activity would not have been easily identified by genomic or metabolomic analyses. It agonizes a non-canonical TLR2–TLR1 heterodimer to release a subset of inflammatory cytokines^9,26,35^. The potency of TLR2 heterodimers is conventionally thought to be governed by a peptide, peptide-like or (poly)saccharide moiety emerging from the dimer interface, and the absence of this chain in a15:0-i15:0 PE might be responsible for the molecule’s unusual immunomodulatory effects (Figs. 1e and 3b)^26,30,32^. Although there is still much to be learned about the pharmacology of a15:0-i15:0 PE, the existing data support a model in which repeated low-level stimulation of the TLR2–TLR1 signalling pathway resets the activation threshold so that weak signals are ignored and strong signals are moderated, thereby contributing to homeostatic immunity^36,37^. It is also important to note that the data underlying the model are from in vitro studies and in vivo studies will be needed to fully validate it. Overall, this study describes the molecular mechanism of a druggable pathway that recapitulates in cellular assays the immunomodulatory effects associated with a prominent member of the gut microbiota.

## Methods

### Bacterial cultivation and extraction of metabolites

*A*. *muciniphila* BAA-835 was inoculated in 3 ml of BHI medium in a 5 ml Falcon tube and incubated under anaerobic conditions at 37 °C for 4  days. Then, 3 ml of the culture was used to inoculate 1 l of BHI medium with 1.5 g of mucin from porcine stomach (Sigma-Aldrich), in a 1 l Pyrex storage bottle (16 bottles × 1 l each, total volume 16  l) and the cultures were incubated for 12 days under anaerobic conditions at 37 °C. After 12 days of static growth, bacterial cultures were centrifuged to separate cell pellets and supernatants (8,000 r.p.m. for 30 min). The cell pellets were extracted with chloroform and methanol (1:1) by stirring for 24 h at room temperature. The solvent mixture was filtered through Whatman qualitative filter paper (grade 3, circle, diameter 125 mm) and dried under vacuum.

For the extraction of supernatants, 100 g of hydrophobic resin mixture (Amberlite XAD4HP and XAD7HP, 20–60 mesh) was added directly to spent media to allow secreted metabolites to adsorb to the resins. Then, the resin mixture containing bacterial metabolites was washed with acetone and methanol (1:1) and stirred for 24 h at room temperature. The solvent mixture with resin mixture was filtered through Whatman qualitative filter paper (grade 3, circle, diameter 125 mm) and concentrated on a rotary evaporator. The cultivation and extraction procedures were repeated eight times (total culture volume, 128 l), yielding 4 g of dry extract from the cell pellets and 15 g of crude extract from the supernatants.

### Bioassay-guided fractionation, purification and identification of a15:0-i15:0 PE

The crude extract from the cell pellets (4 g) was dissolved in chloroform and fractionated by normal-phase chromatography using seven different solvent systems (A, 100% hexane; B, 100% chloroform; C, 100% ethyl acetate; D, 75% ethyl acetate/25% methanol; E, 90% acetone/10% methanol; F, 50% methanol/50% dichloromethane; and G, 100% methanol) with a silica column (Teledyne Isco, RediSep RF Gold Silica 12 g). The pro-inflammatory activity was highly detected in fractions F and G. The mixture of fractions F and G (120 mg and 210 mg, respectively, and 8.3% of total yield) was then subjected to reversed-phase semi-preparative high-performance liquid chromatography (HPLC) (Luna C_8_ (2), 250 × 10 mm, 5 µm) using the following gradient solvent system: 10% methanol/90% water isocratic for 10 min; gradient to 30% methanol/70% water for 10 min; then 30% methanol/70% water to 90% methanol/10% water for 20 min, 90% methanol isocratic for 10 min, gradient to 100% methanol for 25 min; flow rate, 2 ml min^−1^). Fractions were collected every 1 min between 5 min and 75 min, generating 70 fractions. Fractions able to stimulate pro-inflammatory cytokine production from mBMDCs were combined and identified as bacterial PE with BCFAs (22 mg, yield = 0.55%). An essentially pure compound, later identified as a15:0-i15:0 PE, was acquired at a retention time of 63 min (14 mg, yield = 0.35%).

The crude extract from the supernatants (15 g) was dissolved in methanol and filtered through a syringe filter (polytetrafluoroethylene (PTFE), 0.2 µm). The filtered extract was directly injected onto a reversed-phase preparative HPLC column (Luna C_18_ (2), 250 × 21.2 mm, 5 µm) with a gradient mobile solution (30% methanol/70% water to 100% methanol for 30 min, 100% methanol isocratic for 30 min; flow rate, 10 ml min^−1^). Fractions were collected every 2 min from 5 min to 55 min, generating 25 fractions. Fractions able to stimulate pro-inflammatory cytokine production from mBMDCs were collected at 50 min (23.5 mg, 0.16%) and further purified as described above, resulting in additional a15:0-i15:0 (1.2 mg, yield = 0.008%). Overall, 15.2 mg of a15:0-i15:0 was isolated from 19.0 g of crude extract (0.08%).

The structure of a15:0-i15:0 PE was identified by the comprehensive analysis of ^1^H, ^13^C and two-dimensional (2D) NMR spectroscopic data (Extended Data Table 1).

### NMR spectroscopy

All ^1^H NMR spectra were acquired at 500 MHz at 30 °C, and chemical shifts are represented on a δ (beta) scale. Residual protium in the NMR solvent (CDCl_3_, δ 7.26) was used to reference chemical shifts. Data are represented as follows: assignment, chemical shift, integration, multiplicity (s, singlet; d, doublet; t, triplet; q, quartet; m, multiplet; br, broad) and coupling constant in hertz. All ^13^C NMR spectra were obtained at 125 MHz at 30 °C and chemical shifts are represented on a δ scale. The carbon resonances of the NMR solvent (CDCl_3_, δ 77.17) were used to reference chemical shifts. Full assignment of protons and carbons were completed on the basis of the following 2D NMR spectroscopy experiments: gradient ^1^H–^1^H correlation spectroscopy, gradient ^1^H–^13^C heteronuclear single quantum coherence, gradient ^1^H–^13^C heteronuclear multiple bond connectivity. Mnova v.14.2.0 was used to analyse NMR data of natural and synthetic compounds.

### High-resolution mass spectrometry for a15:0-i15:0 PE and other family members

High-resolution mass spectrometry data were collected using Agilent MassHunter Work Station LC/MS Data Acquisition 10.1 and Agilent LC-QTOF Mass Spectrometer 6530 equipped with a 1290 uHPLC system and electrospray ionization detector scanning from *m*/*z* 50 to 3,200. Then 5 μl aliquots of a15:0-i15:0 PE and its family members were injected into a reversed-phase analytical column (Luna C_8_: 100 × 2.1 mm, 5 μm) using a gradient solvent system with 0.1% formic acid (10% methanol/water to 90% methanol/water for 10 min, 90% methanol/water isocratic for 10 min, then gradient to 100% for 10 min; flow rate, 0.3 ml). Agilent MassHunter Qualitative Analysis B.07.00 software was used to analyse the data.

### FA methyl esterification and GC–MS analysis of *A. muciniphila* PE

A 0.1 mg sample of both a15:0-i15:0 PE and complete *A. muciniphila* PE were dissolved in 200 μl of methanol, and 1.4 mg of sodium methoxide was added to prepare a 0.5 mol l^−1^ sodium methoxide solution. The reaction mixture was stirred at room temperature for 3 h then quenched by addition of 1N HCl. The methanolysis products were dried under vacuum and extracted with ethyl acetate and water (300 μl, v/v = 2:1). The water layers were removed, and each of the ethyl acetate layers containing FA methyl esters (FAME) were injected into a gas chromatograph (GC, Agilent MassHunter GC/MS Acquisition B.07.05.2479) combined with a HP-5 ms Ultra Inert column (0.25 mm × 30 m). The temperature of the injector and the detector in the GC was maintained at 150 °C. During analysis, the temperature of the GC column was controlled (150 °C for 3 min, 150–250 °C at 6 °C min^−1^ and 250 °C for 3 min). The FAME derivatives of a15:0-i15:0 PE were composed of i15:0 and a15:0 (1:1 ratio) having retention times at 10.2 min and 9.7 min, respectively. The gas chromatography–mass spectrometry (GC–MS) analysis of FAME derivatives of AmPE displayed i14:0 (15.7%), n14:0 (2.7%), a15:0 (51.7%), i15:0 (23.6%), a16:0 (0.6%), i16:0 (1.8%), a17:0 (1.7%) and a18:0 (2.2%), having retention times at 8.0, 8.6, 9.7, 10.2, 11.3, 11.9, 13.4 and 15.0 min, respectively (Fig. 1d). Agilent MassHunter Qualitative Analysis B.07.00 software was used to analyse GC–MS data.

### *O*-deacylation for determination of a15:0 connected to *sn*-1

A 5 mg sample of a15:0-i15:0 PE was prepared and lyophilized for 24 h. A 1 mg ml^−1^ of NaOMe solution was prepared, and the mixture was dissolved in 500 μl of NaOMe solution at room temperature. The solution was stirred under argon for 30 min. After 30 min, the reaction was quenched by addition of 1N HCl and dried under vacuum. The *O*-deacylated product, a15:0 PE, was purified by reversed-phase HPLC (Luna C_8_ (2): 250 × 10 mm, 5 μm) with an isocratic solvent system (45% acetonitrile/water over 30 min, ultraviolet 210 nm detection, flow rate 2 ml min^−1^). The *O*-deacylated product (1.8 mg) was eluted at 12.5 min, and its structure was determined by one-dimensional and/or 2D NMR spectroscopy (Extended Data Table 2) and by low-resolution electrospray ionization mass spectrometry (ESI-MS) ([M+H]^+^ *m*/*z* at 440; molecular formula, C_20_H_43_NO_7_P).

### Amino-acid feeding experiment

A volume of 5 ml of *A. muciniphila* BAA-835 grown in BHI was inoculated into three 1 l bottles of M9 medium supplemented with 1.5 g of mucin from porcine stomach (Sigma-Aldric) and either 1 mmol l^−1^ of l-leucine, l-isoleucine or l-leucine/l-isoleucine mixture (1:1 ratio) or nothing as a control. The cultures were grown under anaerobic conditions at 37 °C for 12 days. The cell pellets from these cultures were centrifuged and extracted with 40 ml of chloroform and methanol (1:1). The extract was dried under vacuum and dissolved in dimethyl sulfoxide at a 10 mg ml^−1^ concentration and tested for activity in the mBMDC cytokine assay. Statistical significances were determined using an unpaired two-tailed Student’s *t*-test.

### a15:0-i15:0 PE biosynthetic gene identification and analysis

Sequence comparison and analysis of the a15:0-i15:0 PE biosynthetic pathway to the previously reported BCFA biosynthetic pathway and de novo biosynthetic pathway of leucine, isoleucine and valine were performed using blastp (NCBI RefSeq database, updated 8 September 2015), Kyoto Encyclopaedia of Genes and Genomes and Geneious v.11.1.4 for pairwise sequence alignments that were previously reported. The accession number for the genes used in this analysis is CP001071.1.

### Total synthesis for small library of PEs

The total synthesis of a15:0-i15:0 PE, i15:0-a15:0 PE, a15:0-a15:0 PE, i15:0-i15:0 PE and n15:0-n15:0 PE was performed by previously reported methods^38–40^.

### Animal and human-cell studies

Mouse experimental procedures complied with all relevant ethical regulations and were conducted according to protocol 2003N000158 approved by the Institutional Animal Care and Use Committee at Massachusetts General Hospital. Appropriate sample sizes were estimated based on the effect size and variance of cytokine measurements in myeloid cells stimulated with canonical TLR ligands. In all mouse experiments, animals were allocated to experimental groups based on genotype and/or age and sex matched. Male or female wild-type, TLR2^−/−^ or TLR4^−/−^ C57BL/6 mice at least 3–4-weeks old and preferably 7–12 weeks of age were used. Mice were housed with a 12 h light or dark cycle at an ambient temperature of between 18 °C and 24 °C and a relative humidity of between 30% and 70%.

Human monocytes were isolated from buffy coats collected from healthy donors at the Blood Donor Center at Massachusetts General Hospital in compliance with all relevant ethical regulations and according to protocol 2018P001504 approved by the Mass General Brigham Institutional Review Board. Donors provided informed written consent.

### mBMDC cytokine assays

These assays were done as described previously^8^. In brief, femurs and tibias were collected from male or female wild-type, TLR2^−/−^ or TLR4^−/−^ C57BL/6 mice that were at least 3–4-weeks old and preferably 7–12 weeks of age. The bone marrow was pushed from the bones using a needle and syringe of complete Dulbecco’s Modified Eagle Medium (DMEM) supplemented with Gibco GlutaMAX Supplement (35050061), Gibco penicillin-streptomycin (15140122) and 10% heat-inactivated foetal bovine serum (FBS), and strained through a 70 µm nylon filter. The collected bone marrow was then centrifuged, and red blood cells were lysed using Invitrogen eBioscience 1X RBC Lysis Buffer (00-4333-57). The cells were then centrifuged and strained through a 70 µm nylon filter again and resuspended in complete DMEM. Cells were counted and then plated at approximately 5 million cells per plate with approximately 20–40 ng ml^−1^ recombinant murine (Rm) GM-CSF (PreProTech 315-03). They were allowed to grow for 7  days, sometimes with additional feeding of 20–40 ng ml^−1^ RmGM-CSF on day 3. The resulting mBMDCs were then scraped and counted again. They were plated in 96-well tissue culture-treated microplates (Corning CLS3599) from 50,000–90,000 cells per well and allowed to adhere for at least 3 h. The cells were then treated with chromatographic fractions or purified compounds at a final concentration of 50 µg ml^−1^, a final concentration of LPS (InvivoGen tlrl-b5lps) of 3–625 ng ml^−1^ or a final concentration of Pam3CSK4 (InvivoGen tlrl-pms) of 0.250–1.562 µg ml^−1^ and incubated overnight. The following morning, supernatant was removed and an enzyme-linked immunosorbent assay (ELISA) was performed to measure TNFα using an Invitrogen Mouse ELISA kit (88-7324-77) per the manufacturer’s instructions. Gen5 v.3.03 or SoftMax Pro v.6.2.1 (SpectraMax, Molecular Devices) was used to analyse ELISA plates. For cytokine detection using flow cytometry, we used the cytometric bead array mouse inflammation kit from BD Biosciences (552364) per the manufacturer’s instructions. Data were collected with NovoExpress v.1.4.1 and analysed data using FlowJo v.10.7.

### Peripheral blood mononuclear cell cytokine assay

Peripheral blood mononuclear cells (PBMCs) were enriched for monocytes using the RosetteSepHuman Monocyte Enrichment Cocktail (STEMCELL Technologies, catalogue no. 15028). In brief, buffy coats were incubated with monocyte enrichment cocktail for 20 min at room temperature while rocking. They were then diluted with 1X phosphate-buffered saline (PBS) and layered over the Ficoll-Paque PLUS medium (GE Healthcare, catalogue no. 17-1440-02) and centrifuged for 20 min at 1,200*g*. Enriched monocytes were collected and cultured with chromatographic fractions or purified compounds at 50 µg ml^−1^ in DMEM media containing 10% FBS and 1% penicillin-streptomycin. LPS and Pam3CSK4 at a final concentration of 100 ng ml^−1^ were used as controls. After overnight incubation, supernatant was collected and analysed for IL-6, IL-10, IL-12/IL-23p40 and TNFα cytokines using Human Flex Set Kits (BD CBA, catalogue nos. 558276, 558274, 560154 and 560112).

### RNA sequencing

Monocytes were isolated from PBMCs as described previously^41^. Bulk RNA sequencing libraries were prepared using SmartSeq2. Libraries were sequenced on a NextSeq (Illumina). FastQC v.0.11.5 and MultiQC v.1.8 were used to confirm the quality of the sequenced libraries^42,43^. Next, kallisto v.0.46.1 was used with a GRCh38 reference to generate the counts of reads mapped to each gene^44,45^. The matrix of counts was used for the calculation of counts per million (CPM) values, and the generated CPM matrix was treated with log_2_(CPM + 1) to obtain a log expression matrix. A gene with a CPM value greater than 1 was considered as expressed. Samples obtained after the above steps were then used to detect differentially expressed genes via EdgeR v.3.35.1 (ref. ^44^). The lists of differentially expressed genes were generated from likelihood ratio tests based on the generative linear model framework, following the prerequisite gene filtering, normalization and dispersion estimation steps of the software.

### CRISPR targeting

PBMCs were isolated from buffy coats using Sepmate tubes (STEMCELL Technologies) and ammonium–chloride–potassium lysis buffer following the manufacturer’s protocol. Human monocytes were harvested from PBMCs by negative selection using RosetteSep human Monocyte Enriched Cocktail (STEMCELL Technologies) according to the manufacturer’s protocol. Alt-R sgRNAs were purchased from IDT and reconstituted to 100 µmol  l^−1^ with Nuclease-Free Duplex Buffer (IDT). In a sterile polymerase chain reaction strip, the sgRNAs were mixed with Cas9 (IDT, Alt-R S.p. Cas9 Nuclease V3) at a molar ratio of 2:1 (2 µl sgRNA at 100 µmol l^−1^ + 2 µl Cas9 at 5 mg ml^−1^) for each reaction and incubated at room temperature for over 20 min. Monocytes were washed twice with 5 ml of PBS and counted. Then 2 × 10^6^ cells per reaction were resuspended in 16 µl of P3 primary nucleofection solution (Lonza). The 16 µl of cells in P3 buffer was added to each Cas9–ribonucleoprotein complex. The cell–ribonucleoprotein mix was then immediately loaded into the supplied nucleofector cassette strip (Lonza) and nucleofected using 4D-Nucleofector with CM-137 programme. Then 180 µl of prewarmed medium was immediately added into each cassette well. A volume of 1 × 10^5^ cells was seeded into a 96-well plate with medium (RPMI-1640 with 10% FBS, 2 mmol l^−1^ Glutamax, 55 µmol l^−1^ beta-mercaptoethanol, 100 U ml^−1^ penicillin, 100 µg ml^−1^ streptomycin, GM-CSF 800 U ml^−1^ and IL-4 500 U ml^−1^). The medium was changed every 2–3 days. At day 5, MDDCs were stimulated with 10 µg ml^−1^ of *Akkermansia* lipids, 100 ng ml^−1^ of Pam3CSK4, 100 ng ml^−1^ of FSL-1 or 100 ng ml^−1^ of LPS for 18 h or as indicated. Cell supernatants were collected for human TNFα measurements by ELISA (Invitrogen) following the manufacturer’s protocol. SoftMax Pro v.6.2.1 (SpectraMax, Molecular Devices) was used to analyse ELISA plates. The sgRNA sequences used were as follows:

Human TLR1: GGTCTTAGGAGAGACTTATG

Human TLR2: GACCGCAATGGTATCTGCAA

Human TLR6: ATTCATTTCCGTCGGAGAAC

### TLR2–TLR1–a15:0-i15:0 PE complex modelling

Modelling of the a15:0-i15:0 PE ligand complex was based on the crystal structure of the TLR2–TLR1–Pam3CSK4 complex from the Protein Data Bank (PDB ID 2z7x)^28^. The Pam3CSK4 ligand was removed from the crystal structure coordinates, and an a15:0-i15:0 PE ligand was prepared using Lidia and AceDRG in Coot v.0.9 (refs. ^46,47^). The a15:0-i15:0 PE ligand placement in the ligand-binding pockets of TLR2 and TLR1 was guided by the electron density belonging to the acyl chains of the Pam3CSK4 ligand in the crystal structure. Structural figures and videos were generated using ChimeraX v.1.0 (ref. ^48^). Structural biology software was compiled and configured by SBGrid consortium^49^.

### Reporting summary

Further information on research design is available in the Nature Research Reporting Summary linked to this paper.

## Online content

Any methods, additional references, Nature Research reporting summaries, source data, extended data, supplementary information, acknowledgements, peer review information; details of author contributions and competing interests; and statements of data and code availability are available at 10.1038/s41586-022-04985-7.

### Supplementary information

Reporting Summary

Supplementary Video 1 Rotational view of a15:0-i15:0 PE bound to the TLR2–TLR1 receptors.

## Figures and Tables

**Extended Data Table 1:**
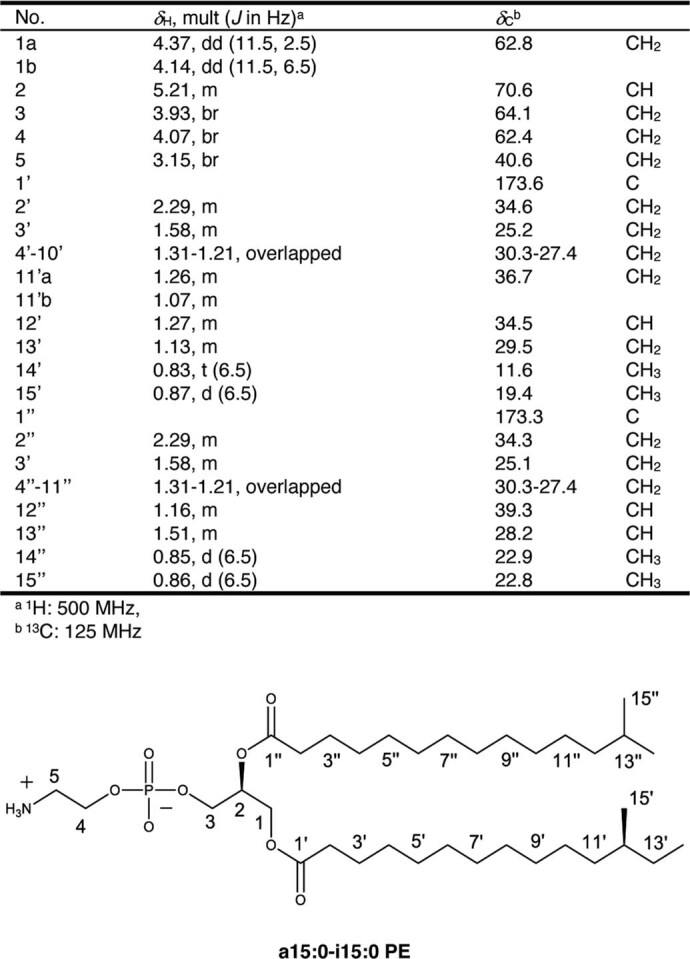
NMR spectroscopic data for identification of the a15:0-i15:0 PE structure

^1^H and ^13^C NMR data (500 MHz) of a15:0-i15:0 PE in CDCl_3_.

**Extended Data Table 2:**
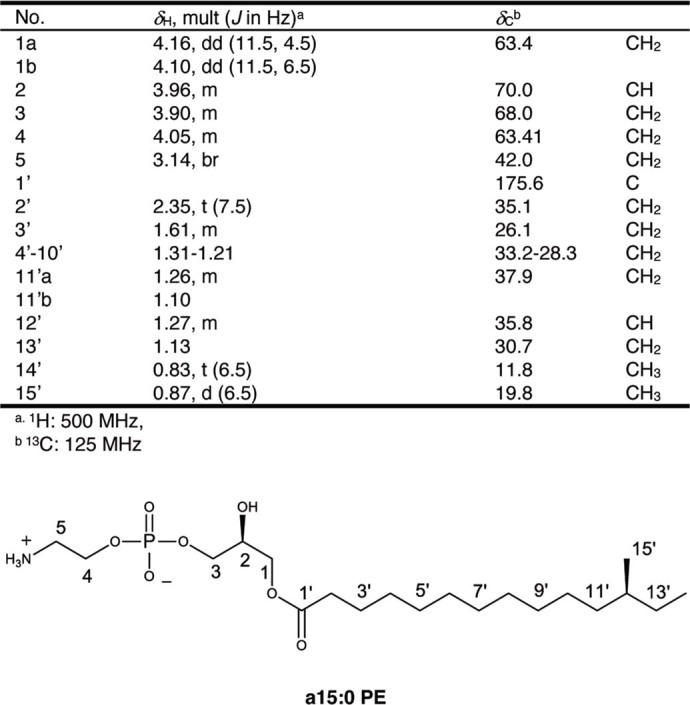
NMR spectroscopic data for identification of the *O*-deacylated product structure

^1^H and ^13^C NMR data (500 MHz) of a15:0 PE in CD_3_OD.

## Data Availability

RNA sequencing data generated during this study are available in the NCBI Gene Expression Omnibus (GEO, GSE199367). NMR data generated during this study are available in Extended Data Tables 1 and 2. Complex modelling was based on the crystal structure from the Protein Data Bank (PDB ID 2z7x). The complete *A. muciniphila* BAA-835 genome was obtained from GenBank (CP001071.1).
