# Supplementary material for: Akkermansia muciniphila phospholipid induces homeostatic immune responses
Source: Nature. 2022 Jul 27;608(7921):168–73. doi: 10.1038/s41586-022-04985-7 (PMC9328018; doi:10.1038/s41586-022-04985-7)
Supplement: Supplementary file 1 — Reporting Summary [file 41586_2022_4985_MOESM1_ESM.pdf]

nature research

Corresponding author(s): Jon Clardy

Last updated by author(s): 3/25/2022

Reporting Summary

Nature Research wishes to improve the reproducibility of the work that we publish. This form provides structure for consistency and transparency in reporting. For further information on Nature Research policies, see our Editorial Policies and the Editorial Policy Checklist.

Statistics

For all statistical analyses, confirm that the following items are present in the figure legend, table legend, main text, or Methods section.

n/a	Confirmed	
☐	☒	The exact sample size (*n*) for each experimental group/condition, given as a discrete number and unit of measurement
☐	☒	A statement on whether measurements were taken from distinct samples or whether the same sample was measured repeatedly
☐	☒	The statistical test(s) used AND whether they are one- or two-sided<br>*Only common tests should be described solely by name; describe more complex techniques in the Methods section.*
☒	☐	A description of all covariates tested
☒	☐	A description of any assumptions or corrections, such as tests of normality and adjustment for multiple comparisons
☐	☒	A full description of the statistical parameters including central tendency (e.g. means) or other basic estimates (e.g. regression coefficient) AND variation (e.g. standard deviation) or associated estimates of uncertainty (e.g. confidence intervals)
☐	☒	For null hypothesis testing, the test statistic (e.g. *F*, *t*, *r*) with confidence intervals, effect sizes, degrees of freedom and *P* value noted<br>*Give P values as exact values whenever suitable.*
☒	☐	For Bayesian analysis, information on the choice of priors and Markov chain Monte Carlo settings
☒	☐	For hierarchical and complex designs, identification of the appropriate level for tests and full reporting of outcomes
☒	☐	Estimates of effect sizes (e.g. Cohen's *d*, Pearson's *r*), indicating how they were calculated

Our web collection on statistics for biologists contains articles on many of the points above.

Software and code

Policy information about availability of computer code

Data collection	Gen5 3.03 or SoftMax Pro 6.2.1 was used to analyze ELISA plates; Agilent Mass Hunter Work Station LC/MS Data Acquisition 10.1 and Agilent LC-QTOF Mass Spectrometer 6530 to collect HRMS data; Agilent Mass Hunter GC/MS Acquisition B.07.05.2479 to collect GC/MS data; and NovoExpress 1.4.1 to collect flow cytometry data
Data analysis	Adobe Illustrator 2020 was used to assemble figures; GraphPad Prism 8 and Microsoft Excel 2016 to perform statistical analyses; Agilent Mass Hunter Qualitative Analysis B.07.00 to analyze HRMS and GC/MS data; FastQC v0.11.5 and MultiQC v1.8 to confirm quality of RNA sequencing libraries; kallisto v0.46.1 and EdgeR v3.35.1 to analyze RNA sequencing data; Geneious 11.1.4 to perform genome analyses; Mnova 14.2.0 to analyze NMR data of natural/synthetic compounds; FlowJo v10.7 to analyze flow cytometry data; Coot 0.9 to model ligand-receptor complex; and ChimeraX 1.0 to generate structural figures

For manuscripts utilizing custom algorithms or software that are central to the research but not yet described in published literature, software must be made available to editors and reviewers. We strongly encourage code deposition in a community repository (e.g. GitHub). See the Nature Research guidelines for submitting code & software for further information.

Data

Policy information about availability of data

All manuscripts must include a data availability statement. This statement should provide the following information, where applicable:

- Accession codes, unique identifiers, or web links for publicly available datasets
- A list of figures that have associated raw data
- A description of any restrictions on data availability

RNA sequencing data generated during this study are available in the NCBI Gene Expression Omnibus (GEO, GSE199367) at https://www.ncbi.nlm.nih.gov/geo/query/acc.cgi?acc=GSE199367. NMR data generated during this study are available in Extended Data Tables 1 and 2. Complex modeling was based on the crystal

structure from Protein Data Bank (PDB ID: 2z7x) at https://www.rcsb.org/structure/2Z7X. The complete A. muciniphila BAA-835 genome was obtained from GenBank (CP001071.1) at https://www.ncbi.nlm.nih.gov/nuccore/CP001071.1.

Field-specific reporting

Please select the one below that is the best fit for your research. If you are not sure, read the appropriate sections before making your selection.

☒ Life sciences ☐ Behavioural & social sciences ☐ Ecological, evolutionary & environmental sciences

For a reference copy of the document with all sections, see nature.com/documents/nr-reporting-summary-flat.pdf

Life sciences study design

All studies must disclose on these points even when the disclosure is negative.

Sample size	Appropriate sample sizes were estimated based on the effect size and variance of cytokine measurements in myeloid cells stimulated with canonical TLR ligands.
Data exclusions	All relevant data were included in this study.
Replication	All experiments in this manuscript were performed at least twice and demonstrated the same or similar results as those published here.
Randomization	In all mouse experiments, animals were allocated into experimental groups based on genotype and/or age- and sex-matched. Human blood samples were obtained from de-identified, volunteer donors.
Blinding	For all biological experiments, investigators performing the experiment were blind to the identities of the samples being tested.

Reporting for specific materials, systems and methods

We require information from authors about some types of materials, experimental systems and methods used in many studies. Here, indicate whether each material, system or method listed is relevant to your study. If you are not sure if a list item applies to your research, read the appropriate section before selecting a response.

Materials & experimental systems

n/a	Involved in the study	
☐	☒	Antibodies
☒	☐	Eukaryotic cell lines
☒	☐	Palaeontology and archaeology
☐	☒	Animals and other organisms
☐	☒	Human research participants
☒	☐	Clinical data
☒	☐	Dual use research of concern

Methods

n/a	Involved in the study	
☒	☐	ChIP-seq
☐	☒	Flow cytometry
☒	☐	MRI-based neuroimaging

Antibodies

Antibodies used	Antibodies were used in immunological assays with mBMDCs using Invitrogen's Mouse TNFalpha Uncoated ELISA kit, catalog # 88-7324.
Validation	All the commercial antibodies are validated by the manufacturers and came with quality assurance statements.

Animals and other organisms

Policy information about studies involving animals; ARRIVE guidelines recommended for reporting animal research

Laboratory animals	Femurs and tibias were collected from male or female wild-type, TLR2-/-, or TLR4-/- C57BL/6 mice at least 3-4 weeks old and preferably 7-12 weeks of age. Mice were housed with a 12-hour light/dark cycle at an ambient temperature between 65-75°F and 30-70% relative humidity.
Wild animals	No wild animals were used in the study.
Field-collected samples	No field collected samples were used in the study.
Ethics oversight	Mouse experimental procedures complied with all relevant ethical regulations and were conducted according to protocol 2003N000158 approved by the Institutional Animal Care and Use Committee (IACUC) at Massachusetts General Hospital.

Note that full information on the approval of the study protocol must also be provided in the manuscript.

Human research participants

Policy information about studies involving human research participants

Population characteristics	This is not applicable to our study. We obtained blood samples from de-identified, volunteer donors in order to isolate human monocytes for in vitro experiments.
Recruitment	Participants were volunteer blood donors.
Ethics oversight	Human monocytes were isolated from buffy coats collected from healthy donors at the Blood Donor Center at Massachusetts General Hospital in compliance with all relevant ethical regulations and according to protocol 2018P001504 approved by the Mass General Brigham Institutional Review Board (IRB). Donors provided informed written consent.

Note that full information on the approval of the study protocol must also be provided in the manuscript.

Flow Cytometry

Plots

Confirm that:

☐ The axis labels state the marker and fluorochrome used (e.g. CD4-FITC).

☐ The axis scales are clearly visible. Include numbers along axes only for bottom left plot of group (a 'group' is an analysis of identical markers).

☐ All plots are contour plots with outliers or pseudocolor plots.

☐ A numerical value for number of cells or percentage (with statistics) is provided.

Methodology

Sample preparation	CBA samples were prepared following the instruction manual of the BD Cytometric Bead Array (CBA) Mouse/Rat Soluble Protein Master Buffer Kit.
Instrument	Agilent NovoCyte Flow Cytometer
Software	NovoExpress 1.4.1, FlowJo v10.7
Cell population abundance	CBA populations were abundant for analysis following the instruction manual of the BD CBA Mouse/Rat Soluble Protein Master Buffer Kit.
Gating strategy	CBA populations were gated following the instruction manual of the BD CBA Mouse/Rat Soluble Protein Master Buffer Kit.

☐ Tick this box to confirm that a figure exemplifying the gating strategy is provided in the Supplementary Information.
